# Targeting of IL-10R on acute myeloid leukemia blasts with chimeric antigen receptor-expressing T cells

**DOI:** 10.1038/s41408-021-00536-x

**Published:** 2021-08-14

**Authors:** Nianci Chen, Yingxi Xu, Junli Mou, Qing Rao, Haiyan Xing, Zheng Tian, Kejing Tang, Min Wang, Jiangxiang Wang

**Affiliations:** 1 State Key Laboratory of Experimental Hematology, Institute of Hematology and Blood Diseases Hospital, Chinese Academy of Medical Sciences & Peking Union Medical College, Tianjin, 300020 China; 2 Tianjin Key Laboratory of Cell Therapy for Blood Diseases, Institute of Hematology and Blood Diseases Hospital, Chinese Academy of Medical Sciences & Peking Union Medical College, Tianjin, 300020 China; 3 National Clinical Research Center for Blood Diseases, Institute of Hematology and Blood Diseases Hospital, Chinese Academy of Medical Sciences & Peking Union Medical College, Tianjin, 300020 China

**Keywords:** Immunotherapy, Cancer therapy

## Abstract

Acute myeloid leukemia (AML) is a biologically and clinically heterogeneous disease with a dismal prognosis and limited treatment options. Chimeric antigen receptor (CAR) T cells have achieved unprecedented clinical responses in patients with B cell malignancies but a dismal consequences in AML. In our previous study, we found that interleukin-10 receptor (IL-10R) was overexpressed in most AML cells, and played an important role in promoting the stemness of leukemia cells. In this study, we developed a novel ligand-based CAR-T cell targeting IL-10R, which displayed striking cytotoxicity both in vitro and in vivo against AML cells. Except for monocytes, it had no significant adverse effects on the normal hematopoietic system, including CD34^+^ hematopoietic stem and progenitor cells (HSPCs). In addition, even though the incorporation of IL-10 in the CAR cassette led to phenotypes change, it had few adverse effects on the survival and biological activity of IL-10 CAR-T cells and did not cause excessive proliferation of leukemia cells. Therefore, we propose IL-10R is a novel promising therapeutic candidate for AML, and IL-10R targeted CAR-T therapy provides a new treatment strategy to improve the prognosis of AML.

## Introduction

Acute myeloid leukemia (AML) is a hematologic malignancy with a poor prognosis. With conventional chemotherapy, the complete remission rate of AML is 60–80% for younger adults and 40–60% for older adults (>60 years), respectively [1]. Together with post-remission therapy (chemotherapy and/or hematopoietic stem cell transplantation), the outcome of AML patients is still frustrating; the 5-year survival rate for young people is about 40%, and for the elderly is even as low as 5–10% [2, 3]. Additional treatment should be provided for patients with dismal outcomes.

Great achievements in CAR-T therapy have exhibited remarkable clinical efficacy against B cell malignancies, especially in acute lymphoblastic leukemia (ALL) [4–6]. However, similar approaches to AML have been challenged. Ongoing efforts to develop CAR-T cells are targeting myeloid lineage antigens such as CD123, CD33, CLL-1, Lewis Y, FLT3, and CD44v6 [7–12]. These CAR-T cells could effectively eradicate malignant blasts in vitro or in vivo. However, CAR-T cells targeting these antigens are always associated with toxicity against the hematopoietic stem and progenitor cells (HSPCs), leading to a risk of myelosuppression or myeloablation [10, 12]. Therefore, in order to effectively utilize CAR-T therapy against AML, potential targets without influencing HSPCs need to be explored.

In our previous study, we found that IL-10 could promote the stemness of AML cells through IL-10R/PI3K/AKT/OCT4 signaling axis, and IL-10RA is essential in promoting the stemness of AML cells (unpublished data). The IL-10R consists of two alpha molecules (IL-10RA) and two beta molecules (IL-10RB). IL-10RA expression is cell-specific, mainly expressed on hematopoietic cells, including B cells, T cells, NK cells, monocytes, and macrophages, generally at a low level [13]. In contrast, IL-10RB is a common receptor and is widely expressed in all kinds of cells [14, 15].

In this study, the natural ligand of IL-10R was utilized as the antigen-binding domain in CAR structure to target IL-10R on the AML cells. The IL-10 CAR-T displayed prominent anti-leukemic effects both in vitro and in vivo, and no obvious cytotoxicity on normal HPSCs. Furthermore, the incorporation of IL-10 in CAR cassette indeed induced phenotype changes of T cells but neither inhibited the survival of CAR-T cells nor caused an excessive proliferation of tumor cells. Thus, we consider IL-10R is a novel promising therapeutic candidate in AML immunotherapy.

## Materials and methods

Methods are provided in the Supplementary Materials and Methods.

## Results

### IL-10R is overexpressed on AML cells and is a prognostic marker

**Fig. 1:**
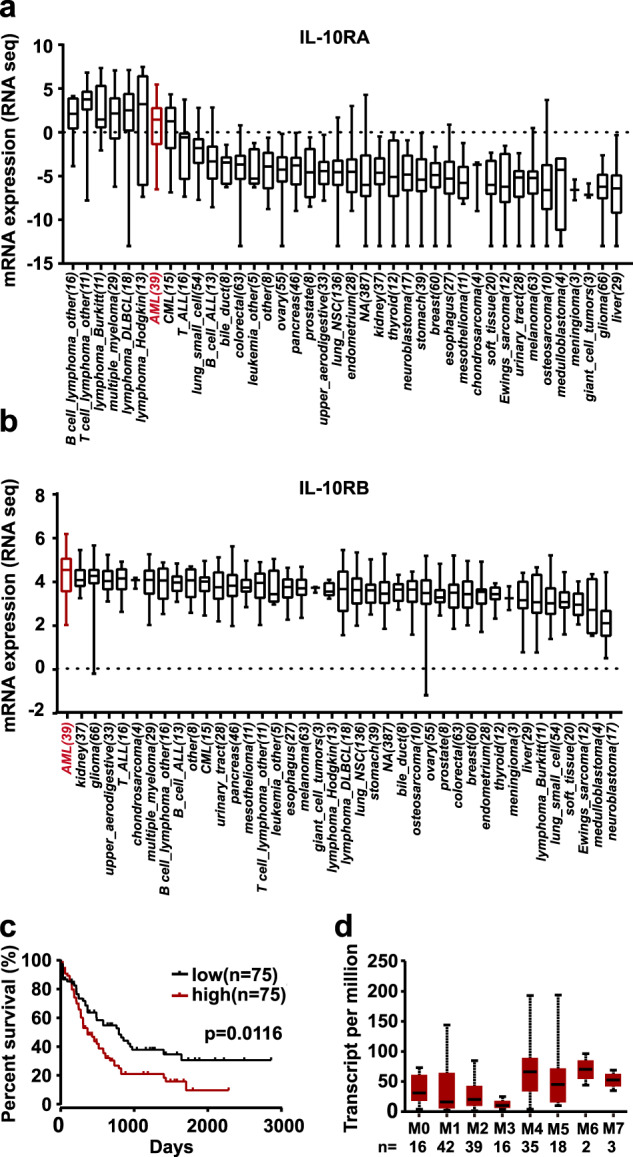
IL-10R is overexpressed on AML cells and is a prognostic marker. **a** The mRNA expression level of IL-10RA in different tumor cell lines was calculated by using the Cancer Cell Line Encyclopedia database. **b** The mRNA expression level of IL-10RB in different tumor cell lines was calculated by using the Cancer Cell Line Encyclopedia database. **c** Kaplan–Meier curves for overall survival of AML patients with the low and high expression level of IL-10RA from the database of TCGA that was analyzed by using OncoLnc online tool (Log-rank test; *n*, number). The percentiles of the low and the high expression group were set to 50%. **d** The mRNA expression level of IL-10RA in AML based on French American British (FAB) classification. This was analyzed by Ualcan database.

In our previous study, IL-10RA was found to overexpress in AML patients compared to that of healthy people (unpublished data). By analyzing the RNA-sequencing data from the CCLE database [16], mRNA expression levels of IL-10RA and IL-10RB were higher in hematological malignant cell lines, including AML (Fig. 1a, b). The OncoLnc online tool (http://www.oncolnc.org/) was used to evaluate the relationship between IL-10RA expression and overall survival (OS) of AML patients. The results showed that patients with the higher expression levels of IL-10RA were associated with a significantly lower OS (Fig. 1c, n = 150, p = 0.0116). Furthermore, Ualcan database [17] was utilized to analyze the expression of IL-10RA in AML patients based on FAB classification, patients with subtype M3 showed mostly lower IL-10RA expression, and M4-M7 displayed higher IL-10RA expression (Fig. 1d). Taken together, it is speculated that IL-10R may be a potential candidate in AML immunotherapy.

### IL-10 CAR-T cells exhibited antigen-specific cytotoxicity against AML cell lines in vitro

**Fig. 2:**
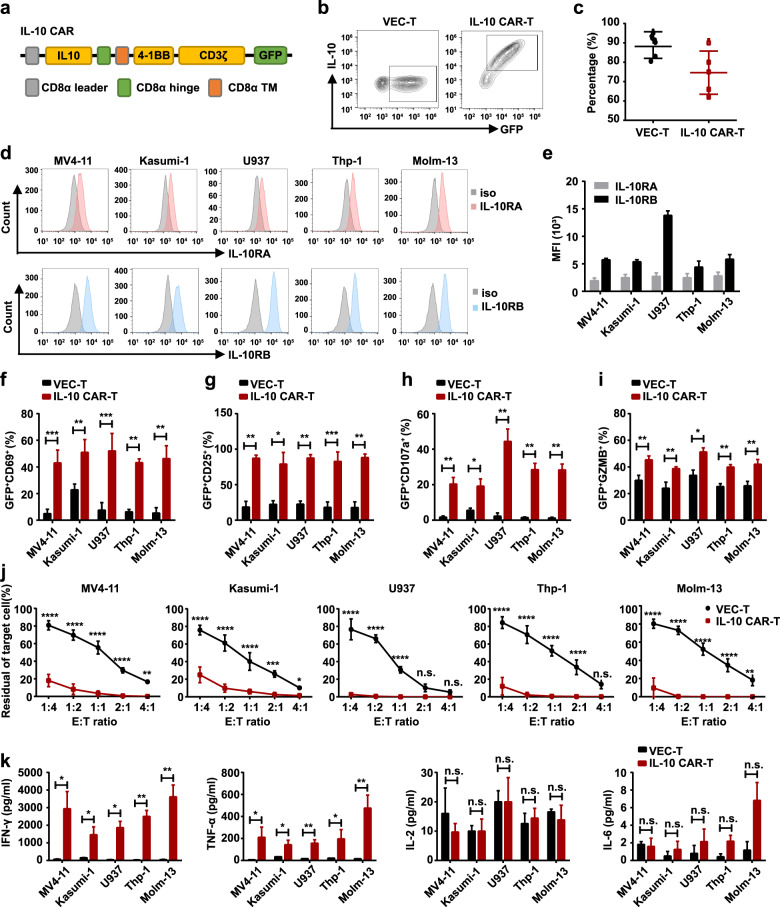
IL-10 CAR-T cells exhibited antigen-specific cytotoxicity against AML cell lines. **a** Schematic diagram of the IL-10 CAR. **b** Representative flow cytometry analysis showing the expression of GFP and IL-10 on VEC-T cells or IL-10 CAR-T cells. **c** Quantification and statistical analysis of the data in (**b**). **d** The expression of IL-10RA (upper panel) or IL-10RB (lower panel) in five leukemia cell lines (MV4-11, Kasumi-1, U937, Thp-1, and Molm-13; iso, isotype control). **e** Quantification and statistical analysis of the mean fluorescence intensity (MFI) of (**d**) (*n* = 3). **f** Quantification and statistical analysis of CD69 expression in VEC-T or IL-10 CAR-T cells (GFP^+^) upon leukemia cells stimulation for 6 h (*n* = 3; **p < 0.01; ***p < 0.001). **g** Quantification and statistical analysis of CD25 expression in VEC-T or IL-10 CAR-T cells (GFP^+^) after 48 h cocultured with leukemia cells (*n* = 3; *p < 0.05; **p < 0.01; ***p < 0.001). **h** Quantification and statistical analysis of CD107a released by VEC-T or IL-10 CAR-T cells (GFP^+^) after 6 h cocultured with leukemia cells (*n* = 3; *p < 0.05; **p < 0.01; ***p < 0.001). **i** Quantification and statistical analysis of Granzyme B (GZMB) released by VEC-T or IL-10 CAR-T cells (GFP^+^) after 6 h cocultured with leukemia cells (*n* = 3; *p < 0.05; **p < 0.01). **j** Effect cells and target cells were cocultured for 48 h at the indicated E:T ratio (1:4, 1:2, 1:1, 2:1, 4:1). Flow cytometry analysis of the percentage of CD3^−^ cells, which represented for the residue of leukemia cell (*n* = 3, two-way ANOVA; n.s. no significant; **p < 0.01; ***p < 0.001; ****p < 0.0001). **k** ELISA data showing the release of INF-γ, TNF-α, IL-2, and IL-6 by VEC-T or IL-10 CAR-T cells after coculture with target cells at the E:T ratio of 1:1 for 48 h (*n* = 3; n.s. no significant; *p < 0.05; **p < 0.01).

The sequence encoding IL-10 mature peptide was cloned in-frame into a lentivirus expression vector containing CAR expression cassettes with 4-1BB and CD3ζ intracellular domains (IL-10 CAR) (Fig. 2a). Transduction efficiencies ranged from 62 to 90% in IL-10 CAR-T and 80 to 92% in VEC-T (Fig. 2b, c). To determine whether IL-10R is an ideal AML target for ligand-based CAR-T therapy, the expression level of IL-10R was evaluated on several AML cell lines by flow cytometry (Fig. 2d). And the mean fluorescence intensity (MFI) of these leukemia cell lines was calculated (Fig. 2e). The MFI of IL-10RA ranged from 1702 to 3372 and that of IL-10RB from 3977 to 13126 in five myeloid leukemia cell lines (MV4-11, Kasumi-1, U937, Thp-1 and Molm-13; Fig. 2d, e). A series of in vitro experiments have been performed to evaluate the efficacy of the IL-10 CAR-T cells. After cocultured with leukemia cells, the expression of activation markers CD69 and CD25 [18] were upregulated in CAR-T cells compared with that in VEC-T cells (Fig. 2f, g). To detect the cytolytic function of T cells, the expression of CD107a and Granzyme B (GZMB) were measured [19]. After 6 h of coculture, higher expressions of CD107a and GZMB were observed in IL-10 CAR-T cells (Fig. 2h, i). After 48 h of coculture, IL-10 CAR-T cells could effectively eliminate leukemia cells at an E:T ratio of 1:1, in some cases, even at an E:T ratio of 1:4 (Fig. 2j). The supernatant of the coculture system was collected to evaluate the cytokine release ability of T cells. The release of Th1 cytokines was significantly increased in IL-10 CAR-T cells, such as IFN-γ and TNF-α, but not IL-2 (Fig. 2k). In addition, IL-6 is considered to be closely related to cytokine release syndrome (CRS) [20] and has not been detected in both VEC-T or CAR-T (Fig. 2k). Overall, IL-10 CAR-T exhibited marked antitumor activity in vitro.

### IL-10 CAR-T cells exhibited antigen-specific cytotoxicity against primary AML cells

**Fig. 3:**
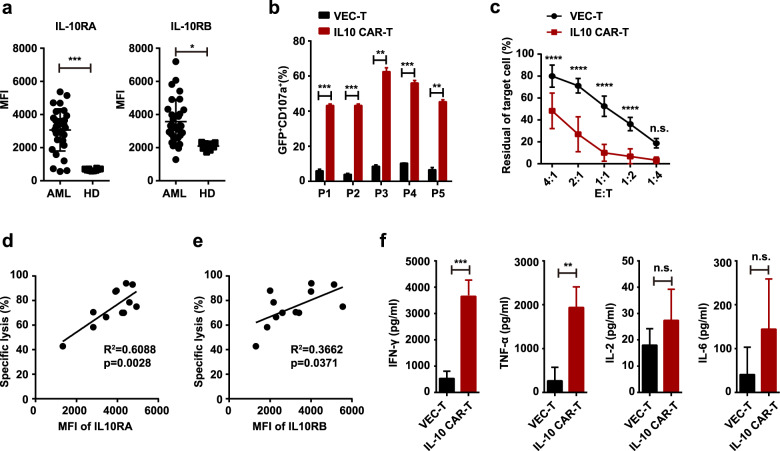
IL-10 CAR-T cells exhibited antigen-specific cytotoxicity against primary AML cells. **a** Quantification and statistical analysis of the expression of IL-10RA and IL-10RB on blast cells from AML patients or bone marrow mononuclear cells (BMMNCs) from healthy donors (HD). **b** Quantification and statistical analysis of CD107a in VEC-T or IL-10 CAR-T cells (GFP^+^) after co-incubated with blast cells at an E:T ratio of 1:1 for 6 h (*n* = 3; **p < 0.01; ***p < 0.001; P, patient). **c** Direct lysis of T cells toward blast cells after cocultured for 48 h at the indicated E:T ratio (1:4, 1:2, 1:1, 2:1, 4:1). Flow cytometry analysis of the percentage of CD34^+^ cells, which represented for the residue of blast cell (*n* = 5, two-way ANOVA; ****p < 0.0001) **d** Correlation analysis between the specific lysis and the level of IL-10RA expression (Spearman correlation analysis, *n* = 12; **p < 0.01). **e** Correlation analysis between the specific lysis and the level of IL-10RB expression (Spearman correlation analysis, *n* = 12). **f** ELISA data showed the cytokines released by T cells after co-incubated with blasts cells for 48 h at the E:T ratio of 1:1 (*n* = 5; **p < 0.01; ***p < 0.001).

To test whether IL-10 CAR-T cells could specifically recognize and kill primary AML cells, we further evaluated the cytotoxicity of IL-10 CAR-T cells against primary leukemia cells. First, the expression of IL-10R on primary AML blasts (*n* = 30) and bone marrow mononuclear cells (BMMNCs) of healthy donors (*n* = 10) was analyzed by flow cytometry. The gating strategy for them was presented in Supplementary Fig. 1a, b. The results indicated that the expression levels of both IL-10RA and IL-10RB were higher on AML blasts than that on healthy donors (Fig. 3a). Since CD34 and CD33 are classic markers for AML, we also detected the expression of these two markers in combination with IL-10R on primary AML samples. The results showed that IL-10R was more frequently expressed on CD33^+^cells (Supplementary Fig. 2b), but its expression was slightly lower on CD34^+^ cells (Supplementary Fig. 2a). In addition, the expression of IL-10R on leukemia stem cells (CD34^+^ CD38^−^) was lower than that on bulk blast cells (Supplementary Fig. 2c).

Subsequently, the CD34^+^ enriched blast cells from five patients were randomly selected as target cells to evaluate the function of CAR-T cells. Consistent with the previous in vitro cytotoxicity assay on AML cell lines, the degranulation ability (Fig. 3b), the specific lysis (Fig. 3c) of IL-10 CAR-T cells were higher than that of VEC-T cells after cocultured with primary blasts. And the specific lysis positively correlated with IL-10R expression (Fig. 3d, e). We also observed the elevated releasing of IFN-γ and TNF-α of IL-10 CAR-T, however, there were no significant differences of IL-2 and IL-6 releasing between IL-10 CAR-T and VEC-T cells (Fig. 3f). These results confirmed the potential clinical utility of IL-10 CAR-T cells in AML patients.

### IL-10 CAR-T exhibited antileukemia effects in vivo

**Fig. 4:**
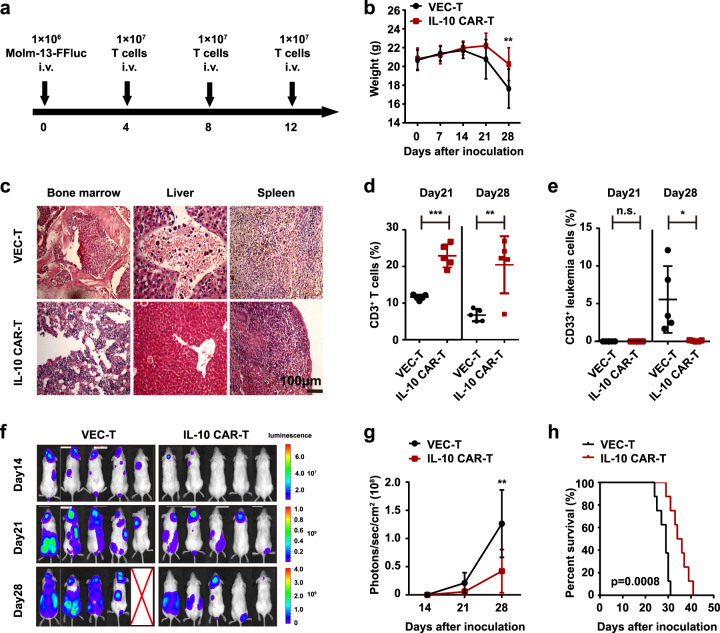
IL-10 CAR-T exhibited anti-leukemia effects in vivo. **a** Schematic diagram of the experimental regimen for validating the efficiency of IL-10 CAR-T. **b** Statistical analysis of body weight of each group measured at indicated days (*n* = 8; two-way ANOVA; *p < 0.05). **c** Pathological analysis of the bone marrow, liver, and spleen of the mice at 21 days after leukemia cells inoculation. **d** Flow cytometry analysis of the proportion of human CD3^+^ T cells in the peripheral blood of mice at day 21 and 28 after leukemia cells inoculation (*n* = 5; **p < 0.01; ***p < 0.001). **e** Flow cytometry analysis of the proportion of human CD33^+^ AML cells in the peripheral blood of mice at day 21 and 28 after leukemia cells inoculation (*n* = 5; n.s. no significant; *p < 0.05). **f** Representative bioluminescence images of each group collected from indicated days. **g** Statistical analysis of the bioluminescence intensity of (**g**) (*n* = 5; *p < 0.05; **p < 0.01; ***p < 0.001). **h** Kaplan–Meier survival curves for mice (*n* = 8; Log-rank test; ***p < 0.001).

To evaluate the efficacy of IL-10 CAR-T cells in vivo, an AML xenograft mice model was established. The NOD/SCID mice were irradiated and injected with 1 × 10^6^ Molm-13-FFluc cells. The regimen of in vivo experiments was shown in Fig. 4a. The body weight of mice decreased in both groups after 21 days and obvious differences could be observed after 28 days of leukemia cells inoculation (Fig. 4b). After 21 days, extensive infiltrations of leukemia cells could be observed in the bone marrow, liver, and spleen of VEC-T group, but only a few infiltrations of leukemia cells in the bone marrow of CAR-T group according to pathological examination (Fig. 4c). The persistence of T cells in peripheral blood or the circulating leukemia cells was evaluated by flow cytometry analysis. Results showed that IL-10 CAR-T cells could persist longer than VEC-T cells in the circulating blood of mice [(11.45 ± 0.3447) vs. (23.04 ± 1.798), day 21; (6.710 ± 0.7415) vs. (20.44 ± 3.483), day 28, *n* = 5, Fig. 4d)]. And in peripheral blood, the proportion of leukemia cells (human CD33^+^) was significantly lower in IL-10 CAR-T treatment group than that of VEC-T treatment group [(5.550 ± 1.982) vs. (0.0780 ± 0.03323), day 28, *n* = 5, Fig. 4e)]. Meanwhile, bioluminescence imaging was used to monitor the growth of leukemia cells in vivo (Fig. 4f). The intensity of bioluminescence signal (photons/s/cm^2^) of the IL-10 CAR-T group was obviously lower than that of the VEC group (Fig. 4g). Median survival times of the IL-10 CAR-T group and VEC-T group were 29 and 35 days, respectively. IL-10 CAR-T could significantly prolong the survival of mice (Fig. 4h). These results demonstrate that IL-10 CAR-T cells are effective for treating AML in vivo.

### IL-10 CAR-T cells have few off-target effects on the hematopoietic system

**Fig. 5:**
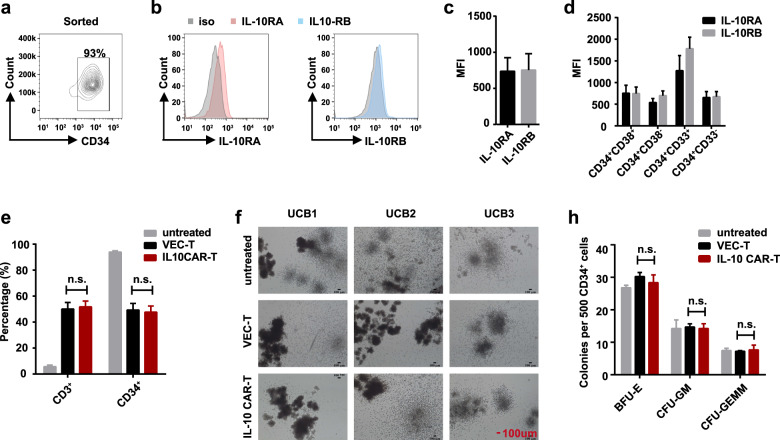
IL-10 CAR-T cells have few off-target effects on the hematopoietic system. Representative flow cytometry analysis showed the proportion of CD34^+^ UBC cells after sorted by magnetic beads. **b** Representative flow cytometry analysis showed the expression of IL-10RA (left panel) and IL-10RB (right panel) on CD34^+^ UCB cells. **c** Quantification and statistical analysis of the data in (**b**) (*n* = 6). **d** Quantification and statistical analysis of the expression of IL-10RA and IL-10RB in different subgroups of cells (progenitor cell, CD34^+^ CD38^+^; granulocyte and monocyte precursor cell; CD34^+^ CD33^+^; hematopoietic stem cell, CD34^+^ CD38^−^). **e** Totally, 5 × 10^4^ CD34^+^ UCB cells which were untreated, or cocultured with 5 × 10^4^ VEC-T or IL-10 CAR-T cells for 24 h. Quantification and statistical analysis of the proportion of CD3^+^ and CD34^+^ cells which were detected by flow cytometry (*n* = 3; n.s. no significant). **f** Representative images showed the colony formation of CD34^+^ UCB cells which were untreated or cocultured with 5 × 10^4^ VEC-T or IL-10 CAR-T cells. **h** Quantification and statistical analysis of the data in (**f**) (*n* = 3; two-way ANOVA; n.s. no significant).

As we mentioned above, IL-10RA is mainly expressed in hematopoietic cells, even though at a low level [13]. For off-target toxicity prediction, we investigated the cytotoxicity of IL-10 CAR-T cells toward the hematopoietic system, including CD34^+^ HSPCs and peripheral blood mononuclear cells (PBMCs). The purity of CD34^+^ umbilical cord blood (UCB) cells was detected after sorting by magnetic beads (Fig. 5a). Then, the expression level of IL-10R on CD34^+^ UCB cells from healthy donors was evaluated. The results indicated lower expression of both IL-10RA and IL-10RB (IL-10RA, 737.5 ± 76.52; IL-10RB, 754.3 ± 92.68; *n* = 6, Fig. 5b, c) on CD34^+^ UCB cells than that on blast cells (IL-10RA, 3069.2 ± 232.10; IL-10RB, 3573.3 ± 244.30, Fig. 3a). Then the CD34^+^ UCB cells were further divided by the expression of CD33 or CD38 [21], the expression of IL-10RA and IL-10RB on myeloid progenitor cells (CD34^+^ CD38^+^), granulocyte and monocyte precursor cells (CD34^+^ CD33^+^) and HSC (CD34^+^ CD38^−^) were shown in Fig. 5d. Due to the low expression of IL-10R on CD34^+^ UCB cells, there were no significant cytotoxic effects of IL-10 CAR-T cells toward CD34^+^ cells compared to that of VEC-T cells (Fig. 5e). At the same time, AML cell lines were used as target cells to confirm the cytotoxic ability of IL-10 CAR-T which could effectively eliminate leukemia cells under the same condition as that of CD34^+^ UCB cells (Supplementary Fig. 3). Then the colony formation assay was performed to assess the differentiative capacity of CD34^+^ UCB cells after cocultured with IL-10 CAR-T cells. After 14 days, the colony morphology was captured and the colony number was counted (Fig. 5f–h). It showed that IL-10 CAR-T did not inhibit the colony formation of CD34^+^ UBC cells, including the formation of burst-forming unit-erythroid, colony-forming unit-granulocyte, macrophage (CFU-GM), and CFU-granulocyte, erythrocyte, macrophage, megakaryocyte (CFU-GEMM) (Fig. 5h). We also investigated the potential cytotoxicity effects on PBMCs. The gating strategy of leukocytes, T cells, B cells, monocytes, and NK cells were shown in Supplementary Fig. 4a, and the expression of IL-10R on these cells was detected (Supplementary Fig. 4b). We cocultured PKH26 labeled PBMCs with VEC-T or IL-10 CAR-T at the E:T ratio of 1:1 for 24 h. The results showed that only monocytes were vulnerable and targeted (Supplementary Fig. 4c). In contrast, IL-10 CAR-T cells did not kill leukocytes, B cells, T cells, and NK cells (Supplementary Fig. 4c). Together, these results suggested that IL-10 CAR-T had few side effects on the hematopoietic system.

### The effects of IL-10 on the survival, phenotype, and biological activity of IL-10 CAR-T cells

**Fig. 6:**
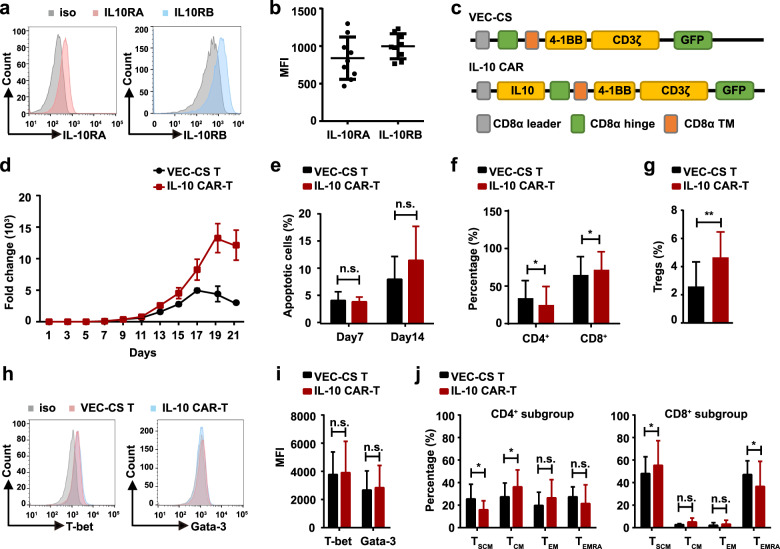
The effects of IL-10 on the survival, phenotype, and biological activity of IL-10 CAR-T cells. Representative flow cytometry analysis of the expression of IL-10RA (left panel) and IL-10RB (right panel) on T cells. **b** Quantification and statistical analysis of the MFI in (**a**) (*n* = 9). **c** Schematic diagram of the VEC-CS and IL-10 CAR constructs. **d** The proliferation of VEC-CS T or IL-10 CAR-T cells cultured for 21 days (*n* = 3). **e** Quantification of the apoptosis of VEC-CS T or IL-10 CAR-T cells at day 7 and day 14 (*n* = 3; two-way ANOWA; n.s. no significant). **f** Quantification and statistical analysis of the proportion of CD4^+^ or CD8^+^ T cells at day 14 (*n* = 9; *p < 0.05). **g** Quantification and statistical analysis of the proportion of CD4^+^ CD25^+^ FOXP3^+^ Tregs at day 7 (*n* = 5; **p < 0.01). **h** Representative flow cytometry analysis showing the expression of T-bet (left panel) and GATA-3 (right panel) on T cells. **i** Quantification and statistical analysis of the MFI in (**h**) (*n* = 3; n.s. no significant). **j** Quantification and statistical analysis of the proportion of T_SCM_ (CD45RA^+^ CCR7^+^), T_CM_ (CD45RA^+^ CCR7^−^), T_EM_ (CD45RA^−^ CCR7^+^), and T_EMRA_ (CD45RA^+^ CCR7^−^) subgroups in VEC-CS T or IL-10 CAR-T cells. CD4^+^ T cell analysis was shown in the left panel, and CD8^+^ T cell analysis was shown in the right panel (*n* = 9; two-way ANOWA; n.s. no significant; *p < 0.05).

In our study, a ligand-based anti-IL-10R CAR-T cell was investigated. IL-10, the ligand of IL-10R, is a dimeric cytokine with both immunosuppressive and immunostimulatory activities [22–24]. Although IL-10 CAR-T did not cause a cytotoxic effect on T cells (Supplementary Fig. 2c), the IL-10 in the CAR construct, which may still interact with the IL-10R on T cells during the long period in vitro culture, leading to changes in phenotypes or biological functions of CAR-T cells. The expression of IL-10RA and IL-10RB on T cells of healthy donors was shown in Fig. 6a, b (IL-10RA, 838.7 ± 93.87; IL-10RB, 997.7 ± 55.31; *n* = 9). In order to investigate the effect of IL-10 signaling on CAR-T cells, another control vector with the same structure as CAR, but without IL-10 was generated (VEC-CS, Fig. 6c). Firstly, the ex vivo long-term survival (up to 21 days) of IL-10 CAR-T was assessed. It indicated that IL-10 did not disrupt the proliferation of CAR-T cells (Fig. 6d). In both VEC-CS and IL-10 CAR-T cells, they could expand 50- to 100-fold on day 7 and about 3000-fold on day 14. On the other hand, there was no difference in the proportion of apoptotic cells between VEC-CS or IL-10 CAR-T cells when cultured for 7 days [(4.100 ± 0.8785) vs. (3.527 ± 0.1468); *n* = 3; Fig. 6e; left panel)] and 14 days [(7.817 ± 2.334) vs. (11.17 ± 3.469); *n* = 3; Fig. 6e; right panel)]. Then the phenotype of IL-10 CAR-T cells was analyzed. It showed that the proportion of CD8^+^ T cells was increased (Fig. 6f). This may be because IL-10 inhibits the proliferation and cytokines synthesis of CD4^+^ T cells, but does not directly affect CD8^+^ T cells [25, 26]. In order to further explore the effect of IL-10 on the phenotypes of CD4^+^ CAR-T cells, the proportion of regulatory T cells (Tregs), type 1T helper (Th1) cells and type 2T helper (Th2) cells were analyzed. Tregs characterized by the specific expression of the transcription factor forkhead box P3 (FOXP3) are generally regarded as the immunosuppressive subgroup of CD4 ^+ ^T cells [27]. Flow cytometry analysis demonstrated that, compared with VEC-CS T cells, the percentage of Tregs in IL-10 CAR-T cells was upregulated [(2.593 ± 1.007) vs. (4.653 ± 1.046), *n* = 3, Fig. 6g)]. To determine whether IL-10 induces the shift of Th1 and Th2 cells, T-bet and GATA3, the key transcription factor of Th1 and Th2 [28, 29], were tested, and it showed that there was no difference in T-bet or GATA-3 expressions between VEC-CS T cells and IL-10 CAR-T cells (Fig. 6h, i). Finally, the differentiation status of VEC-CS T cells and IL-10 CAR-T cells were evaluated through the expression of CD45RA or CCR7 [30, 31]. The results indicated that, in CD4^+^ T cells, the proportion of stem cell memory (T_SCM_, defined as CD45RA^+^ CCR7^+^) subset was lower in IL-10 CAR-T cells than that in VEC-CS T cells. On the contrary, compared to CD8^+^ VEC-CS T cells, CD8^+^ IL-10 CAR-T exhibited a slightly higher proportion of T_SCM_ subset and a lower proportion of terminal differentiation effector cells (T_EMRA_, defined as CD45R^+^ CCR7^−^) (Fig. 6j). This indicated that IL-10 was superior at preserving the stem-like properties of CD8^+^ T cells [32–34], and the increase in the proportion of CD8^+^ T_SCM_ can improve the antitumor potency of CAR-T cells [35]. Taken together, these studies demonstrated that even though the incorporation of IL-10 in the CAR cassette led to phenotypic changes, it had few adverse effects on the survival and biological activity of IL-10 CAR-T cells.

### IL-10 did not facilitate the proliferation of leukemia cells

**Fig. 7:**
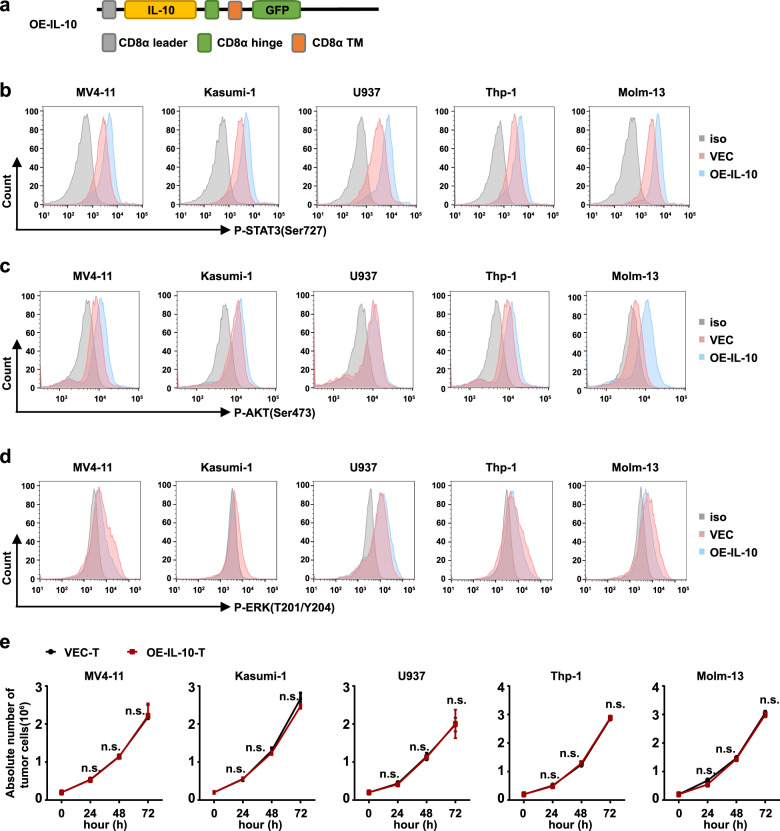
IL-10 did not facilitate the proliferation of leukemia cells. **a** Schematic diagram of the construct of OE-IL-10. **b**–**d** Leukemia cells were cocultured with VEC-T or OE-IL-10-T cells for 3 h at the E:T ratio of 1:1. **b** Representative flow cytometry analysis showing the phosphorylation of STAT3 at Tyr705 in leukemia cells. **c** Representative flow cytometry analysis showing the phosphorylation of AKT at Ser473 in leukemia cells. **d** Representative flow cytometry analysis showing the phosphorylation of ERK at T202/Y204 in leukemia cells. **e** Quantification of the proliferation of leukemia cells after cocultured with VEC-T or OE-IL-10-T cells within 72 h (the absolute number of leukemia cells were counted by cell density × volume × the ratio of CD3^-^ cells; two-way ANOVA; n.s. no significant).

Apart from the effects on CAR-T cells, the ligand-based CAR may also activate the signal pathway of leukemia cells and induce proliferation of them [21]. Therefore, a vector comprising IL-10, the hinge and transmembrane domain, but without the intracellular signal domain, was established (OE-IL-10) (Fig. 7a). After coculture VEC-T cell or OE-IL-10-T cell with leukemia cells for 3 h, compared with VEC-T group, the leukemia cells treated with OE-IL-10-T cells induced a slight increase in the phosphorylation of STAT3 and AKT (Fig. 7b, c), and a mild decrease in the phosphorylation of ERK (Fig. 7d). No matter what, OE-IL-10-T cells did not promote the proliferation of leukemia cells within 72 h (Fig. 7e). And from Figs. 2j and 3c, the results showed that IL-10 CAR-T could eliminate leukemia cells within 48 h. Therefore, it indicated that leukemia cells were killed by IL-10 CAR-T cells before they were induced to proliferate.

### The increased IL-10 signaling may lead to the tumor microenvironment reprogramming

**Fig. 8:**
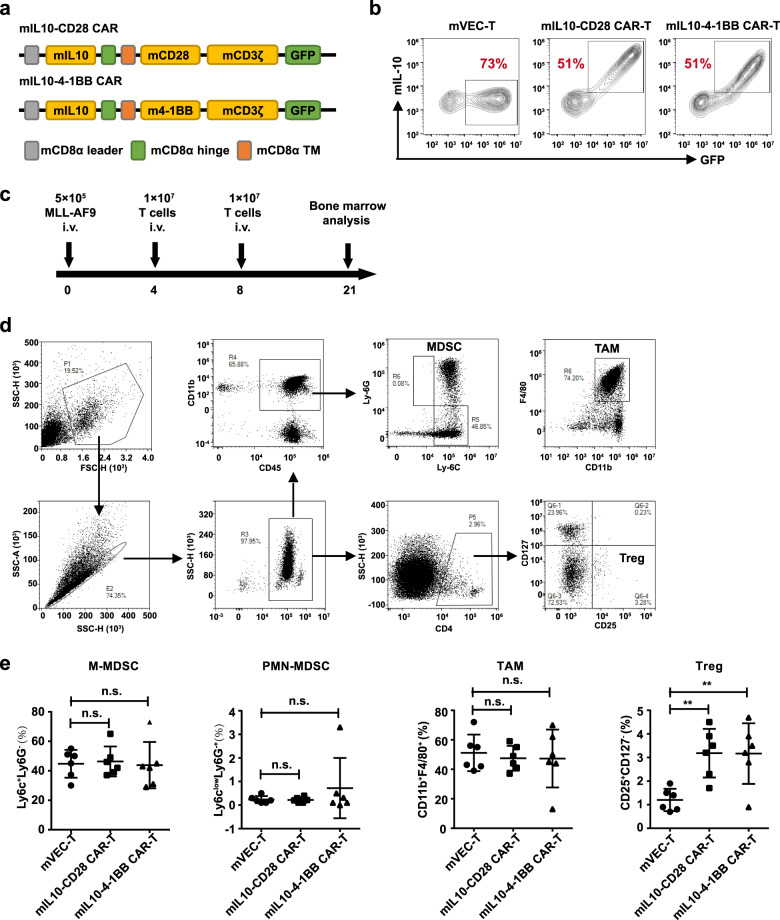
The increased IL-10 signaling may lead to the tumor microenvironment reprogramming. **a** Schematic diagram of the murine IL-10 CAR (structures containing murine IL-10 followed by the murine CD8α hinge and transmembrane domain, intracellular CD28 or 4-1BB costimulatory domain, and intracellular CD3ζ signaling domain. **b** Representative flow cytometry analysis showing transduction efficiency of mVEC-T, mIL-10-CD28 CAR-T, or mIL10-4-1BB CAR-T cells. **c** Schematic diagram of the experimental regimen. **d** The gating strategy of flow cytometric analysis showing the method to distinguish immunosuppressive cells in BM microenvironment (M-MDSC, monocytic-myeloid derived suppressor cells; PMN-MDSC, polymorphonuclear-myeloid derived suppressor cells; TAM, tumor associated macrophage). **e** Quantification and statistical analysis of the proportion of M-MDSC, PMN-MDSC, TAM, and Treg (CD45^+^ CD11b^+^ Ly6C^+^ Ly6G^−^, M-MDSC; CD45^+^ CD11b^+^ Ly6C^low^ Ly6G^+^, PMN-MDSC; CD45^+^ CD11b^+^ F4/80^high^, TAM; CD4^+^ CD25^+^ CD127^−^, Treg; n.s. no significant; **p < 0.01).

The therapeutic effect of IL-10 CAR-T has been performed with xenograft model in immunodeficient mice in Fig. 4. Finally, we sought to comprehensively evaluate the effects of IL-10 on the tumor microenvironment (TME) with a murine IL-10 based CAR-T in an immunocompetent MLL-AF9 model. The murine CAR constructs comprising murine IL-10, murine CD8α hinge and transmembrane domain, the costimulatory domain of CD28 or 4-1BB, and the signaling domain of CD3ζ were shown in Fig. 8a. Transduction efficiencies are around 50% in mIL10-CD28 or mIL10-4-1BB CAR-T cells and 70% in mVEC-T cells (Fig. 8b). The C57BL/6 mice received 4.5 Gy of sublethal total body irradiation and were injected with 5 × 10^5^ MLL-AF9 cells. The regimen of in vivo experiments was shown in Fig. 8c. Twenty-one days after leukemia cells inoculation, mice were euthanized and analyzed for the immunosuppressive cells in the bone marrow. Analysis of endogenous immune infiltrates revealed there were no differences of monocytic myeloid-derived suppressor cells (M-MDSC); polymorphonuclear (PMN) MDSC; tumor-associated macrophage (TAM) between the mVEC-T group and mIL10-CD28 or mIL10-4-1BB CAR-T groups. However, in mIL10-CD28 and mIL10-4-1BB CAR-T treated mice, the proportion of Tregs was increased (Fig. 8d). Thus, the IL-10 based CAR-T structure, due to the increased IL-10 signaling, has a risk of reprogramming an immunosuppressive TME.

## Discussion

Our previous study revealed that IL-10RA is overexpressed in most AML cells and played an essential role in promoting the stemness of leukemia cells. Moreover, we found that the higher expression of IL-10RA correlated with a less favorable prognosis in AML (Fig. 1c). These results indicate that IL-10RA could be used as a biomarker and a potential target for AML therapeutic interventions. Encouraged by the impressive clinical developments of CAR-T therapy in B cell malignancies, especially in CD19 CAR-T treating ALL [5, 6], the ligand-based anti-IL-10R CAR-T cells (IL-10 CAR-T) were developed. To our knowledge, it is the first report of utilizing IL-10R as a therapeutic target in AML CAR-T therapy. Furthermore, according to the database results in Fig. 1, we found that the higher expression of IL-10R is not restricted in AML but also in other hematological malignancies. To verify it, the expression of IL-10RA and IL-10RB on other kinds of tumor cell lines including Burkitt lymphoma (Raji, Daudi), ALL (Nalm-6), multiple myeloma (H929, MM1.S) (Supplementary Fig. 5a, b) was analyzed, and IL-10 CAR-T exhibited obvious cytotoxicity towards these cell lines (Supplementary Fig. 5c). Therefore, IL-10 CAR-T has potential applications in the treatment of a broad of hematological malignancies, not only AML.

The basic design of CARs consisted of three major components: an antigen-binding domain, a hinge, and transmembrane domain, and an intracellular signaling domain. Among these, the antigen-binding domain is the extracellular portion of the CAR, which recognizes the target antigen and determines the specificity of CAR-T cells. Traditionally, the antigen-binding domains of CARs have been composed of the variable heavy and variable light chains of monoclonal antibodies, which are connected by a flexible linker to form a single-chain variable fragment (scFv). However, an scFv sequence with appropriate antigen-binding affinity can not always be obtained [36]. The binding property of ligand with receptor makes it feasible to use ligand as antigen recognition domain in CAR structure. Many CARs based on ligand or receptor are being tested in preclinical and clinical studies in a variety of malignancies, including FLT3 ligand to target FLT3^+^ AML [21], a proliferation-inducing ligand (APRIL) to target multiple myeloma expressing B cell maturation antigen [37], granulocyte macrophage colony-stimulating factor (GM-CSF) to target the GM-CSF receptor (CD116) of juvenile myelomonocytic leukemia [38] or natural killer cell receptor D (NKG2D) to target NKG2D ligands on the surface of malignant hematologic cells [39]. In our study, the natural ligand of IL-10R was utilized as the antigen-binding domain in CAR structure to target IL-10R on the surface of AML cells and showed potential efficacy. However, by the introduction of a ligand or receptor molecule in CAR structure, relevant signaling in CAR-T cells or tumor cells would be activated. During our experiments, even though the incorporation of IL-10 in CAR cassette didn’t inhibit the survival of CAR-T cells nor cause excessive proliferation of tumor cells, the increased proportion of Treg during CAR-T cell culture in vitro (Fig. 6g) and in the microenvironment in vivo was observed (Fig. 8e), which may dampen the therapeutic effect and clinical outcome [40]. Therefore, in order to be further applied to the clinic, the scFv based IL-10R CAR-T should be developed to avoid the induction of the immunosuppressive microenvironment.

During CAR-T therapy, one of the major concerns is the possibility of the widespread expression of the target antigen, which may lead to on-target, off-tumor toxicity [41]. Given that IL-10R expresses in various hematologic cells, a series of experiments were performed to detect the possibility of cytotoxicity on normal cells. First, the cytotoxicity of IL-10 CAR-T cells on CD34^+^ HSPCs was examined. Unlike the expression of IL-10R on leukemia cell lines or primary leukemia cells, the expression of IL-10A or IL-10RB on CD34^+^ UCB cells was much lower. As a result, after cocultured for 24 h at an E:T ratio of 1:1, there was no obvious cytotoxicity of IL-10 CAR-T cells on CD34^+^ UCB cells. Then the off-target effects on PBMCs were explored, it showed that IL-10 CAR-T did not exhibit cytotoxicity towards T cells, B cells, or NK cells, but most monocytes were targeted after coculture, thus the influence of IL-10 CAR-T on monocytes need further study. As for T cells, even though the expression of IL-10R on normal T cells was detected, it did not cause CAR-T cell fratricide (Supplementary Fig. 4c and Fig. 6d, e), which was observed in CD5 CAR-T or CD7 CAR-T therapy toward T cell malignancy [42, 43]. We speculated that it might be due to the far lower expression of IL-10RB in T cells than that in AML cell lines or primary leukemia cells. In contrast to IL-10RA, IL-10RB alone was unable to bind IL-10. Only after IL-10 binding to IL-10RA, the conformation of cytokine changed, then the association of IL-10/IL-10RA complex with IL-10RB occurred [44]. The affinity of IL-10 to the IL-10R complex is markedly higher (500–620 pM) than to the isolated IL-10RA (50–250 pM) [45]. Hence, we consider the surface expression of IL-10RB is of vital importance in IL-10′ binding. And the positive correlation between specific lysis and the expression of IL-10RB (Fig. 3e) also proved it. However, more evidence is required to verify its safety to avoid the on-target, off-tumor toxicity in IL-10 CAR-T therapy.

Another common and severe toxicity in CAR-T cell therapy is CRS or CAR-T related encephalopathy syndrome, which is caused by multiple cytokines release from the CAR-T cells and other immune cells [46]. Many inflammatory cytokines, especially IL-1, IL-6, IL-8, GM-CSF, macrophage inflammatory protein-1α (MIP-1α), and monocyte chemoattractant protein-1 (MCP-1) are responsible for CRS [47–50]. IL-10 is a cytokine with multifaceted biological effects, it sheds effects on various cell populations, such as T cells, B cells, NK cells, monocytes, and macrophages [13]. To the latest knowledge, it appears that monocyte/macrophages are the main target cells of the IL-10 inhibitory effects, IL-10 inhibits the functions of monocytes/macrophages [51] thus depresses releasing pro-inflammation mediators such as IL-1β, IL-6, GM-CSF, and G-CSF [13]. In clinical practice, the utility of tocilizumab to block the IL-6 signaling pathway can be used to relieve the symptoms of CRS. From the results of Supplementary Fig. 4c we speculate that the cytotoxicity of IL-10 CAR-T on monocytes, to some extent, would alleviate the severity of CRS.

Overall, even though the ligand-based IL-10 CAR-T cells led to potential immunosuppressive effects on TME, we still demonstrated that IL-10R is a potential target for AML since IL-10R targeted CAR-T therapy displayed significant anti-AML effects both in vitro and in vivo, and it displayed little cytotoxicity on normal hematopoietic cells. Targeting IL-10R by CAR-T therapy may be a promising approach for the treatment of AML.

## Supplementary information

Supplementary Figures

Supplementary Materials and Methods

check lists
