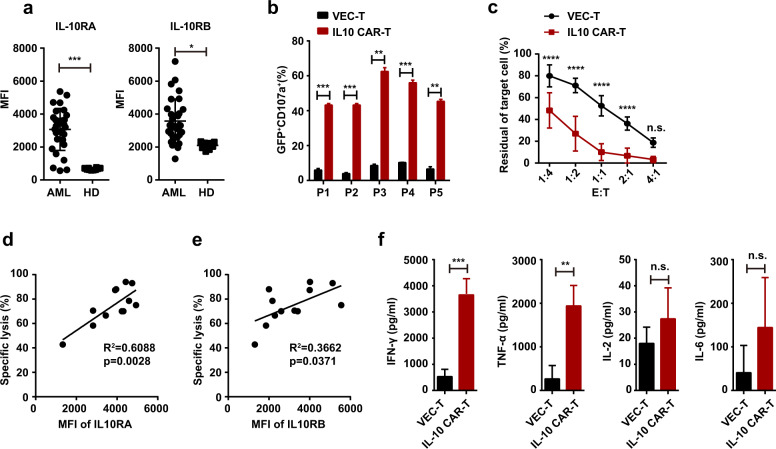# Correction: Targeting of IL-10R on acute myeloid leukemia blasts with chimeric antigen receptor-expressing T cells

**DOI:** 10.1038/s41408-022-00627-3

**Published:** 2022-03-16

**Authors:** Nianci Chen, Yingxi Xu, Junli Mou, Qing Rao, Haiyan Xing, Zheng Tian, Kejing Tang, Min Wang, Jianxiang Wang

**Affiliations:** 1 State Key Laboratory of Experimental Hematology, Institute of Hematology and Blood Diseases Hospital, Chinese Academy of Medical Sciences & Peking Union Medical College, Tianjin, 300020 China; 2 Tianjin Key Laboratory of Cell Therapy for Blood Diseases, Institute of Hematology and Blood Diseases Hospital, Chinese Academy of Medical Sciences & Peking Union Medical College, Tianjin, 300020 China; 3 National Clinical Research Center for Blood Diseases, Institute of Hematology and Blood Diseases Hospital, Chinese Academy of Medical Sciences & Peking Union Medical College, Tianjin, 300020 China

**Keywords:** Immunotherapy, Cancer therapy

Correction to: *Blood Cancer Journal* 10.1038/s41408-021-00536-x, published online 14 August 2021

Due to our negligence, an unintentional error appeared in Figure 3c. The E:T ratio should be listed as 1:4 1:2 1:1 2:1 4:1 instead of 4:1 2:1 1:1 1:2 1:4 in abscissa. This may cause confusion among readers. We are so sorry for not noting the error and have enclosed the revised Figure 3 in supplemental file 1.