# Supplementary material for: Targeting of IL-10R on acute myeloid leukemia blasts with chimeric antigen receptor-expressing T cells
Source: Blood Cancer J. 2021 Aug 14;11(8):144. doi: 10.1038/s41408-021-00536-x (PMC8364556; doi:10.1038/s41408-021-00536-x)
Supplement: Supplementary file 1 — Supplementary Figures [file 41408_2021_536_MOESM1_ESM.docx]

**Supplementary Figures**


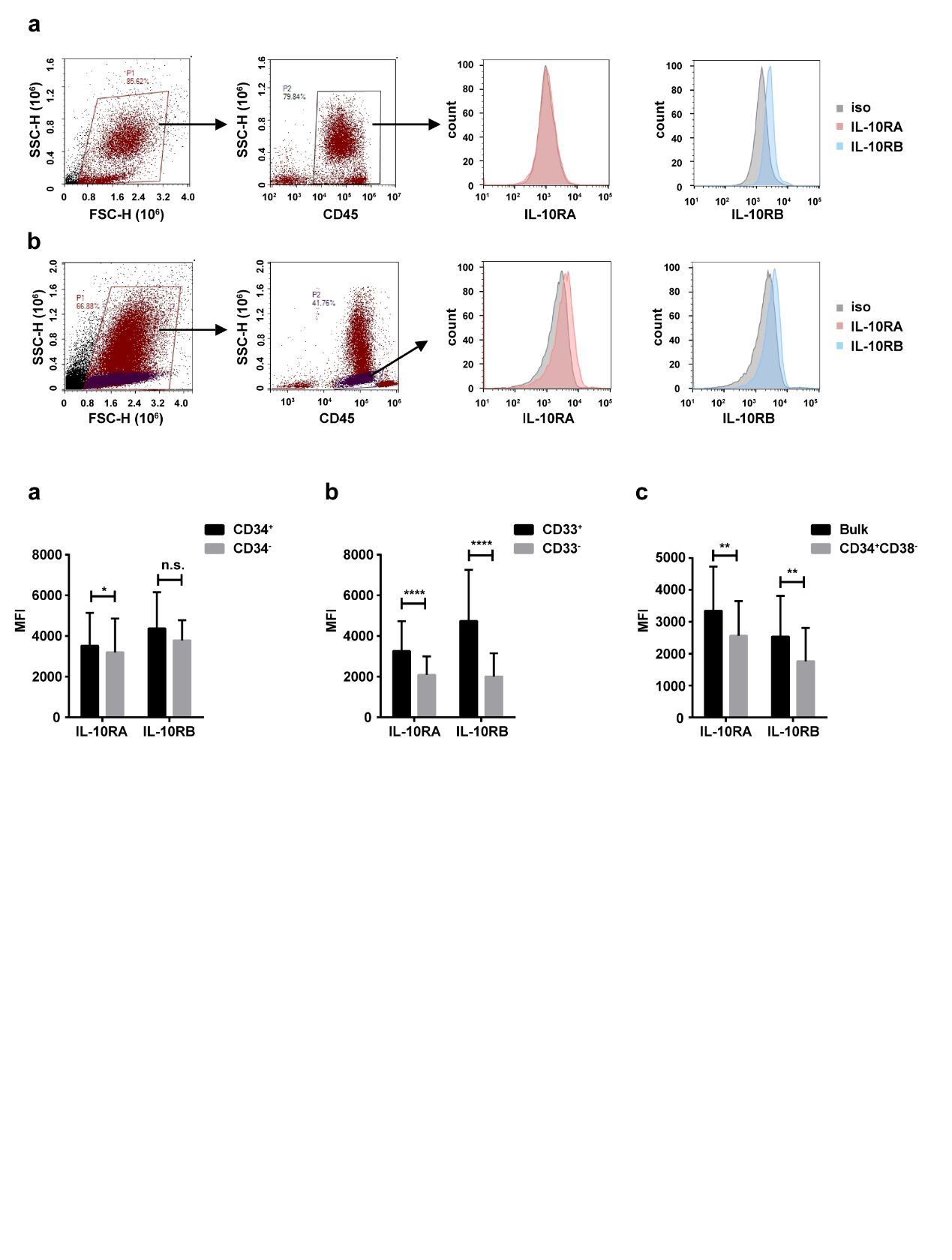


**Supplementary Figure 1. The gating strategy for AML blast cells or healthy BMMNCs**

1. The gating strategy and the expression of IL-10RA/IL-10RB on BMMNCs from healthy donors. BMMNCs were initially gated based on forward and side scatter properties. Subsequently, CD45^+^ BMMNCs were gated.
2. The gating strategy and the expression of IL-10RA/IL-10RB of AML blasts. BMMNCs were initially gated based on forward and side scatter properties. Subsequently, AML blasts were gated based on CD45^dim^ and SSC^low^.


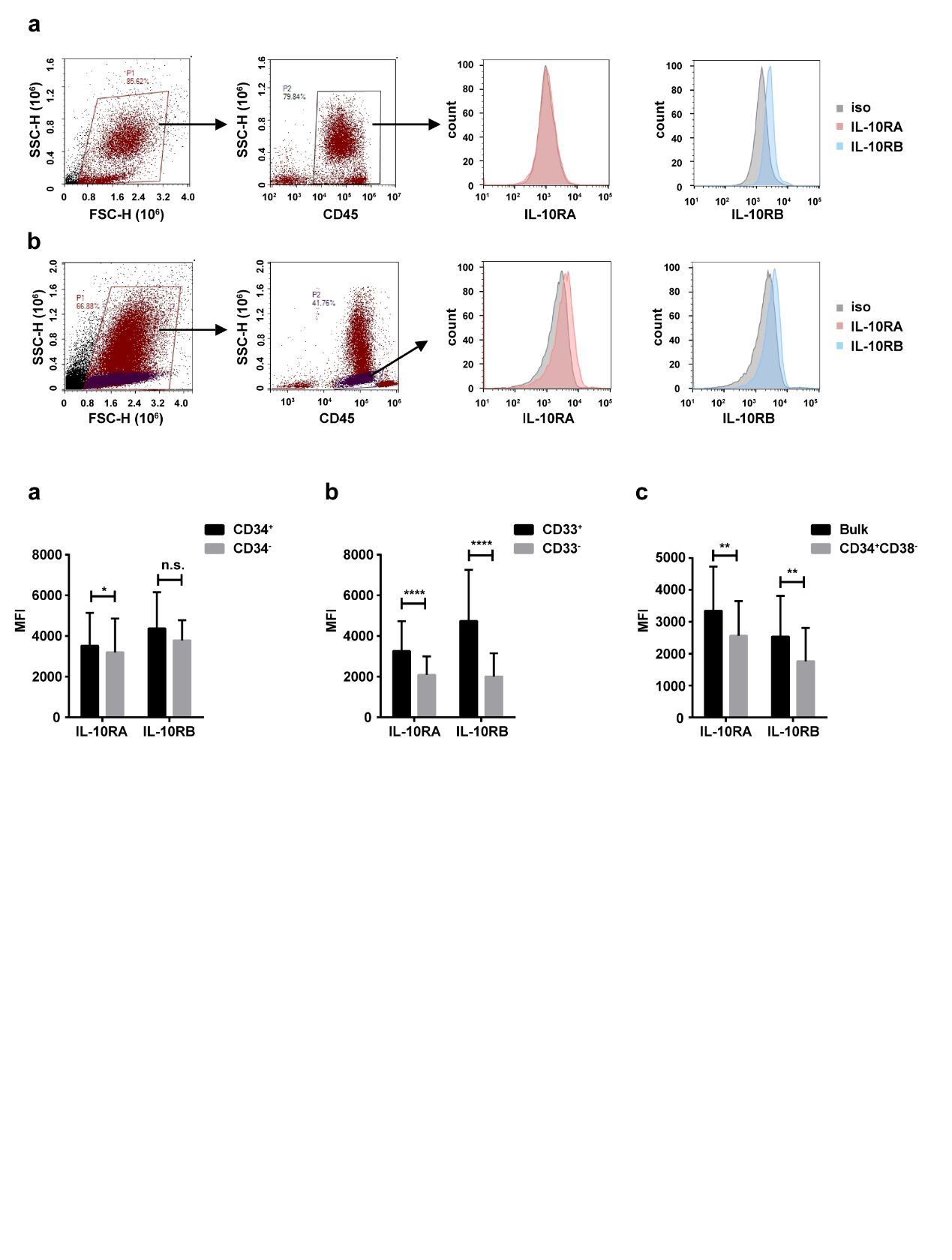


**Supplementary Figure 2. The expression of IL-10RA and IL-10RB with other AML markers**

1. IL-10RA and IL-10RB expression on CD34^+^ AML blast cells compared to that on CD34^-^ AML blast cells (n=15; *, p < 0.05; n.s., no significant).
2. IL-10RA and IL-10RB expression on CD33^+^ AML blast cells compared to that on CD33^-^ AML blast cells (n=24; ****, p < 0.0001).
3. IL-10RA and IL-10RB expression on CD34^+^CD38^-^ LSCs compared with bulk BMMNCs (n=15; **, p < 0.01).


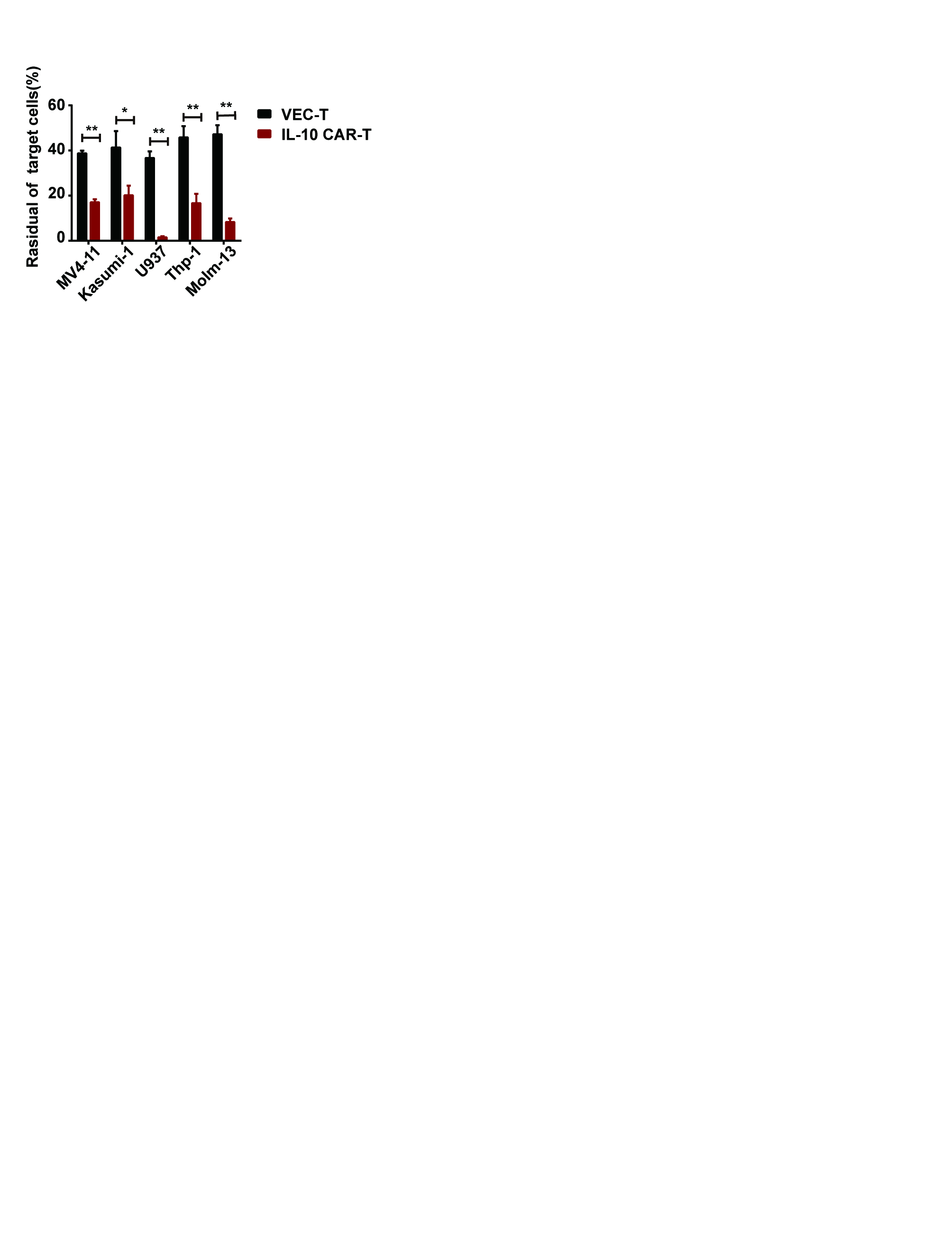


**Supplementary Figure 3. Effectively lysis of IL-10 CAR-T cells toward AML cell lines**

5×10^4^ VEC-T or IL-10 CAR-T cells were cocultured with target cells at an E:T ratio of 1:1 for 24 hours. Flow cytometry analysis of the percentage of CD3^-^ cells (n=3; *, p < 0.05; **, p < 0.01).


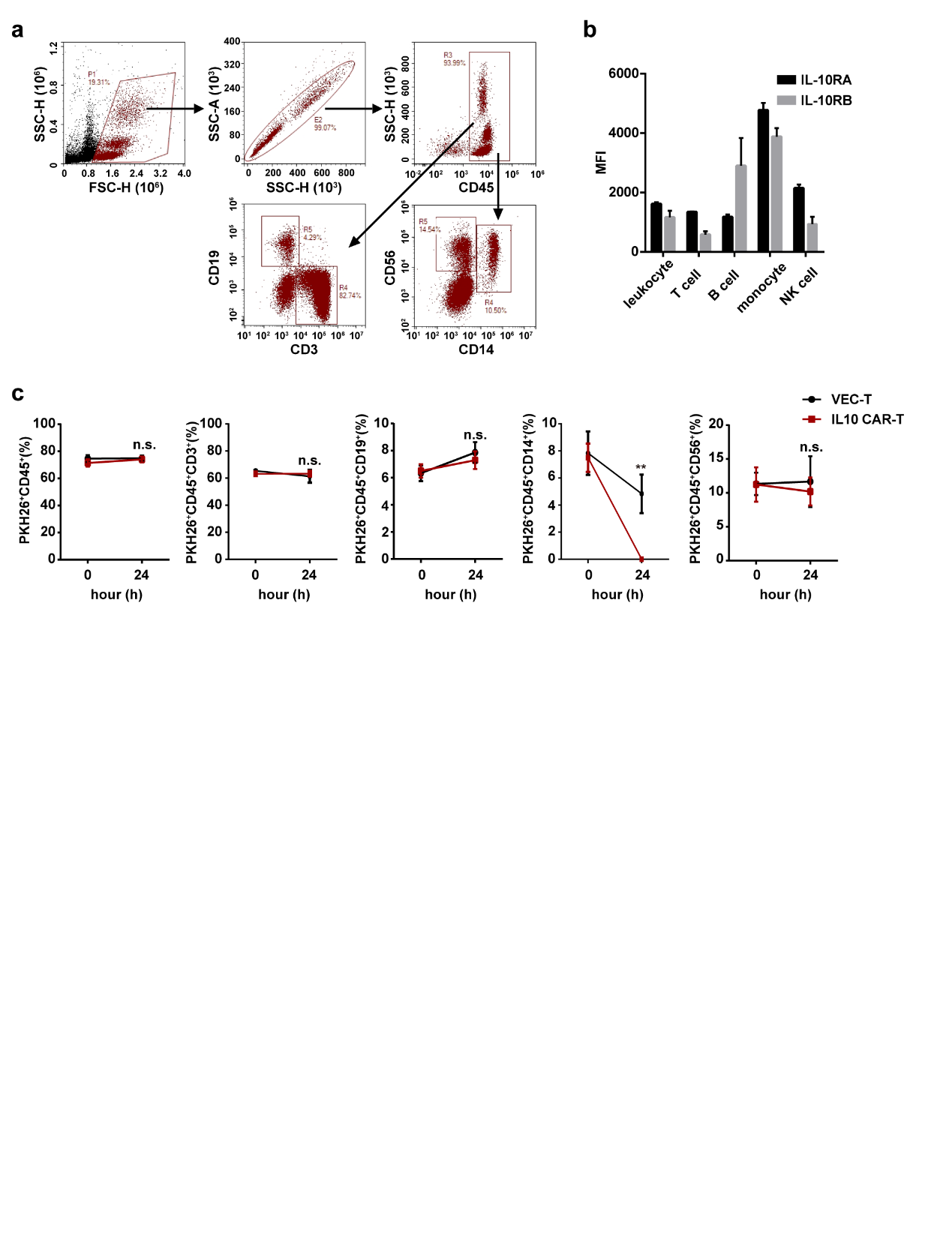
**Supplementary Figure 4. Except for monocytes, IL-10 CAR-T did not show cytotoxicity toward peripheral immune cells.**

1. The peripheral blood mononuclear cells (PBMCs) from healthy donors were isolated by Ficoll gradient density centrifugation. Specific markers allowed discrimination of the subpopulation as leukocyte (CD45^+^), T cell (CD45^+^CD3^+^), B cell (CD45^+^CD19^+^), monocyte (CD45^+^CD14^+^), and NK cell (CD45^+^CD56^+^).
2. The expression of IL-10RA and IL-10RB on peripheral immune cells.
3. PBMCs was labelled with red fluorescent cell linker PKH26, then cocultured with VEC-T or IL-10 CAR-T at the E:T ratio of 1:1 for 24h. Cytotoxicity of IL-10 CAR-T or VEC-T was measured with flow cytometry by specific markers for each group (leukocyte, PHK26^+^CD45^+^; T cell, PHK26^+^CD45^+^CD3^+^; B cell, PHK26^+^CD45^+^CD19^+^; monocyte, PHK26^+^CD45^+^CD14^+^; NK cell, PHK26^+^CD45^+^CD56^+^; n=3; n.s., no significant; **, p < 0.01).


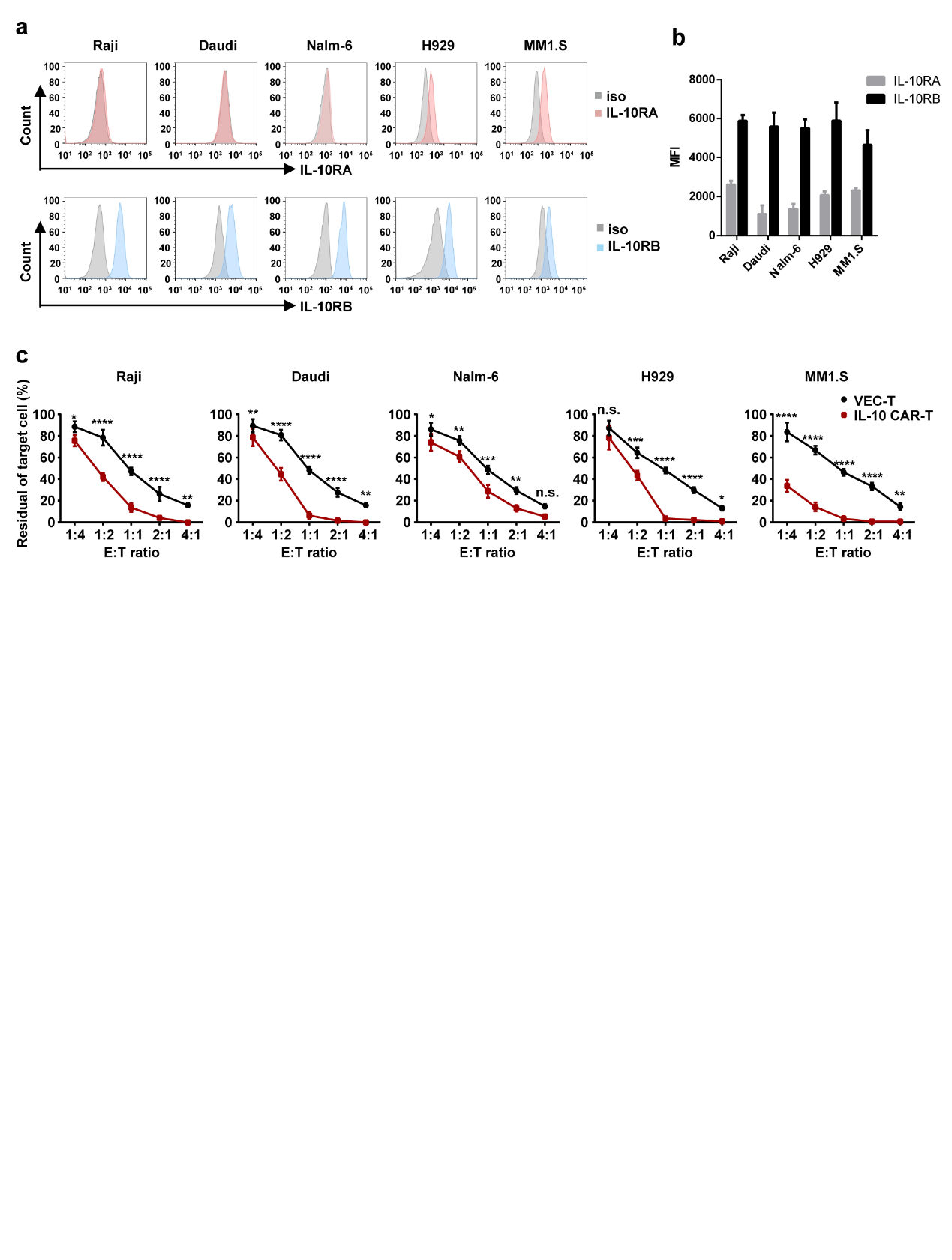


**Supplementary Figure 5. The cytotoxicity of IL-10 CAR-T toward other hematologic cell lines.**

1. The expression of IL-10RA (upper panel) or IL-10RB (lower panel) in other hematologic cell lines (Raji, Daudi; Nalm-6, H929 and MM1.S).
2. Quantification and statistical analysis the mean fluorescence intensity (MFI) of the data in a (n=3).
3. VEC-T or IL-10 CAR-T cocultured with target cells for 48h at the indicated E:T ratio (1:4, 1:2, 1:1, 2:1, 4:1). Flow cytometry analysis of proportion of CD3^-^ cells (n=3; Two-way ANOWA; n.s., no significant; *, p < 0.05; ***, p < 0.001; ****, p < 0.0001).
