# Supplementary material for: Targeting of IL-10R on acute myeloid leukemia blasts with chimeric antigen receptor-expressing T cells
Source: Blood Cancer J. 2021 Aug 14;11(8):144. doi: 10.1038/s41408-021-00536-x (PMC8364556; doi:10.1038/s41408-021-00536-x)
Supplement: Supplementary file 2 — Supplementary Materials and Methods [file 41408_2021_536_MOESM2_ESM.docx]

**Supplementary** **Materials and Methods**

**Primary tumor and cancer cell line database**

Cancer Cell Line Encyclopedia (CCLE) database was used to calculate IL-10R expression across different tumor cell lines ^1^. The prognosis associated with the IL-10R was analyzed using the OncoLnc online analysis tool, which combines prognostic data from The Cancer Genome Atlas (TCGA) database with mRNA expression levels. The percentiles of the low and the high expression group were set to 50%. The expression data of IL-10R in AML based on French American British (FAB) classification was obtained from the Ualcan database ^2^.

**Primary cells from AML patients and healthy donors**

Bone marrow (BM) samples of newly diagnosed AML patients and healthy donors were obtained from the Institute of Hematology and Blood Diseases Hospital. Peripheral blood of healthy donors was obtained from Tianjin Blood Center. Human umbilical cord blood (UCB) was obtained from Pathological Cell Bank of the Institute of Hematology and Blood Diseases Hospital. All subjects were under informed consent which in accordance with the ethical guideline (declaration of Helsinki) and proved by the ethical advisory board of the Institute of Hematology and Blood Diseases Hospital.

**Cell culture**

293T cells were maintained in Dulbecco’s modified Eagle’s medium (DMEM, Gibco, USA) supplemented with 10% FBS and 2mM glutamine (Cat.# 25030081, Gibco, USA). U937, THP-1, MOLM13, and Kasumi-1 cells were grown in RPMI-1640 (Gibco, USA) with 10% FBS. MV4-11 cells were maintained in Iscove’s modified Dulbecco’s medium (IMDM, Gibco, USA) supplemented with 10% FBS. Primary AML cells were cultured in IMDM supplemented with 15% FBS, 100ng/ml rhFLT3-L (Cat.# 300-19, PeproTech, USA), 100ng/ml rhSCF (Cat.# 300-07, PeproTech, USA) and 50ng/ml rhTPO (Cat.# 300-18, PeproTech, USA). Human T cells were cultured in lymphocyte medium KBM581 (Corning, USA) supplemented with 5% FBS and 50IU/mL rhIL-2 (Cat.# 200IL-500, RD systems, USA).

Primary murine T cells cultured in RPMI 1640-Glutamax medium supplemented with 10% FBS, 50IU/ml hIL-2, 10ng/ml rhIL-7 (Cat.# 200-07, PeproTech, USA), 10ng/ml rhIL-15 (Cat.# 200-15, PeproTech, USA), 50μM β-mercaptoethanol (Cat.# 21985023, Gibco, USA), and 10mM nonessential amino acids (Cat.# 11140050, Gibco, USA).

All cells were tested on a regular basis for mycoplasma and were negative.

**Plasmid construction and lentivirus production**

*For human vector construction*

Using PCR and restriction sites BamH I and EcoR I, the full length of IL-10 mature peptide was cloned from cDNA of human T cells as binding domain and subsequently inserted to the second-generation CAR with PCDH-EF1α backbone which was constructed before ^3^. The PCR primers for IL-10 mature peptide are as follows: Forward: 5’- GGATCCAGCCCAGGCCAGGGCACCC-3’; Reverse: 5’-GAATTCG TT TCGTATCTTCATTGT CATGTAG-3’.

The empty vector pCDH-EF1α-T2A-GFP (System Biosciences, SBI) was used as control (VEC).

Another control vector using the same structure as CAR but without antigen binding domain named VEC-CS. The PCR primers used to amplify the segment from the hinge domain to the co-stimulation domain are as follows: Forward: 5’-GGATCCACCACGACGCCAGCGCCGCGACCA-3’; Reverse: 5’-GAATTCCTAGCGAGATCC GGTGGAGCCGGG-3’.

To explore whether the ligand-based CAR activate the signal pathway of target cells and induce the proliferation of target cells, the vector included membrane-bound IL-10 in the absence of intracellular signal domain was constructed, named OE-IL-10. The PCR primers used to amplify the segment from leader sequence to transmembrane domain are as follows: Forward: 5’-AGAGCTAGCGCCACCATGGCCTTACCA-3’; Reverse: 5’-AGCGGCCGCGTAAAGGGTGATAACCAGTGACAG-3’

*For murine vector construction*

The retroviral vector MSCV-IRES-GFP was used as the backbone for murine CAR constructs. The constructs of murine CAR including murine IL-10 followed by murine CD8α hinge and transmembrane region, the endo-domain of murine CD28 or 4-1BB and CD3ζ (mIL10-CD28 or mIL10-4-1BB) ^4, 5^.

All lentivirus containing corresponding vectors were produced in 293T cells. Viral supernatants were harvested after 24 and 48 h, filtered through 0.45μm filter, then centrifuged using high-speed ultra-centrifugation at 50000g for 2h. Then the virus pellet was resuspended in 1ml KBM581 for human T cell infection, and in 1ml RPMI 1640-Glutamax medium for murine T cell infection.

**Human T cell isolation, activation and infection**

The detailed protocol of human CD3^+^ T cell isolation and activation has been described previously ^6^. After activation for 24h, T cells were transduced with lentiviral supernatants and replaced with fresh culture medium after 48h.

**Murine T cells isolation, activation and infection ^5^**

Murine T cells isolated from the spleen of C57BL/6 mice using the EasySep Mouse T cells Isolation Kit (Cat.#19851, Stem cell technologies, Canada).

1×10^6^/ml T cells were plated in 24-well plate in RPMI 1640-Glutamax medium supplemented with 10% FBS and stimulated with Dynabeads® Mouse T-Activator CD3/CD28 (Cat.# 11452D, Gibco, USA) at a bead-to-cell ratio of 1:1 and 50IU/ml hIL-2. Non-treated 24-well plates were precoated with Retronectin (Cat.# T100A, Takara Bio, Japan) at a final concentration of 20 ug/ml, overnight at 4°C. Twenty-four hours later, Retronectin-precoated plates were washed with PBS, and blocked with 2% BSA in PBS for 30 min at room temperature (RT). Subsequently, to prepare the virus-coated-plates, they were washed twice with PBS, lentivirus was added, then plates were spun with 2000g for 2h at RT. Then, the activated T cells transferred to the coated 24-well plate. The plates were further centrifuged for 10 min at 300g. The cell mixture was replenished with fresh T cell medium after 24h and maintained at a cell density of 0.5 to 10^6^ cells/ml.

Transduction efficiency was determined on day 7 with a flow cytometry using anti-human or anti-mouse IL-10 antibody and GFP reporter gene.

**Flow cytometry assay**

All antibodies used in the following flow cytometry analysis were purchased from Biolegend.

*For cell surface markers detection*

Anti-human antibodies used for surface markers are anti-IL-10 (JES3-9D7), anti-IL-10RA (3F9), anti-IL-10RB (S17009F), anti-CD3 antibody (OKT3), anti-CD4 (OKT4), anti-CD8 (SK1), anti-CD25 (BC96), anti-CD69 (FN50), anti-CCR7 (G043H7), anti-CD45RA (HI100), anti-CD33 (WM53), anti-CD34 (561), anti-CD45 (HI30) and anti-CD38 (HIT2).

All anti-mouse antibodies used in flow cytometry analysis including: anti-CD45 (S18009F), anti-CD3 (17A2), anti-CD4 (RM4-5), anti-CD25 (PC61), anti-CD127 (SB/199), anti-CD11b (M1-70), anti-CD11c (N418), anti-Ly-6C (HK1.4), anti-Ly6-G (1A8), anti-MHCII (M5/114.15.2), anti-F4/80 (BM8).

Cells were collected and washed once with PBS prior to the addition of antibodies and then incubated for 30 minutes on ice in the dark, then washed once prior to analysis.

*For intracellular markers staining*

Cells were stained with surface antibodies first, then cells were washed, fixed (Cat.# 420801, 426803, Biolegend, USA), permeabilized (Cat.# 424401, 425401, Biolegend, USA), and stained with intracellular proteins’ antibody following the manufacturer's instructions. Antibodies used for intracellular markers are FOXP3 (206D), T-bet (4B10), GATA-3(16E10A23), p-STAT3 (Tyr705) (13A3-1), p-AKT (Ser473) (SDRNR,), p-ERK (T202/Y204) (MILAN8R).

*For cell proliferation analysis*

The absolute counting of cells was analyzed by flow cytometry with dead cells and debris excluded.

*For cell apoptosis detection*

T cells were stained with Alexa Fluor 647 AnnexinV and PI (Cat.# 640912) according to the manufacturer’s instruction.

All flow cytometric analyses were performed on the FACS Novocyte 2060R (Agilent, USA).

**Degranulation assay**

*For CD107a staining*

1×10^5^ VEC-T or CAR-T cells were cocultured with equal number of target cells in a 96-well plate in the presence of 2μM Monensin (Cat.# 420701, Biolegend, USA) and anti-human CD107a antibody (H4A3) in 200μl medium. After 6 h, cells were harvested, and stained with anti-human CD3 antibody.

*For Granzyme B staining*

5×10^5^ VEC-T or CAR-T cells were cocultured with target cells with the E:T ratio of 1:1 in 24-well plate. 5μg/ml Brefildin A (Cat.# 420601, Biolegend, USA) and 2μM Monensin were added at the same time. After 6 h, cells were collected and stained with anti-human CD3 antibody first, and then fixed, permeabilized and stained with anti-human Granzyme B antibody (QA16A02).

**CAR-T specific killing assay**

*For cell lines*

2×10^5^ CAR-T or VEC-T cells were cocultured with different numbers of target cells at E:T ratios of 4:1, 2:1, 1:1, 1:2 and 1:4 in 24-well plate containing 1ml of T cells growth medium without rhIL-2. After coculture for 24, 48 or 72 h, cells were collected and quantified by flow cytometry based on CD3 expression. The percentage of CD3^-^ cells represented the level of residual target cells.

*For primary AML cells*

Bone marrow mononuclear cells (BMMNCs) from AML patients were isolated through Ficoll density centrifugation. Blast cells were gated from CD45^dim^SSC^low^ and analyzed for the expression of IL-10R by flow cytometry. BMMNCs was subsequently sorted by human CD34 MicroBead Kit (Cat.# 130046702, Miltenyi Biotec, Germany). The enriched CD34^+^ blast cells cocultured with IL-10 CAR-T or VEC-T cells in a 24-well plate at E:T ratios of 4:1, 2:1, 1:1, 1:2 and 1:4. The residual target cells were detected as the percentage of CD34^+^ cells.

*For HPSCs*

CD34^+^ HSPCs in human UCB were isolated by human CD34 MicroBead Kit (Cat.# 130046702, Miltenyi Biotec, Germany). 5×10^4^ UCB CD34^+^ cells were cocultured with equal numbers CAR-T or VEC-T in 1ml serum-free stem cell medium (Cat.# 09605, STEM CELL, Canada). After 24h, cells were collected to detect the residual CD3^+^ T cells or CD34^+^ UCB cells by flow cytometry.

**Cytokine release assay**

After 48 h, the co-cultured supernatant was collected to detect the release of cytokines by ELISA. The concentrations of human IL-2 (Cat.# S2050), IFN-γ (Cat.# SIF50C), TNF-α (Cat.# STA00D), and IL-6 (Cat.# S6050) were measured according to the manufacturer's instructions (R&D Systems, USA)

**In vivo murine experiments**

All animal experiments were performed in compliance with the animal care guidelines approved by the Institutional Animal Care and Use Committees of the State Key Laboratory of Experimental Hematology.

*For human IL-10 CAR-T in vivo*

Female NOD/SCID mice of 6-8 weeks old were purchased from Institute of Laboratory Animal Sciences (CAMS&PUMC, China). Mice were irradiated at 1.5Gy and intravenously inoculated with 1×10^6^ Molm-13 cells which were transduced with firefly-luciferase (Molm-13-FFluc). On day 3, mice were allocated to VEC-T and IL-10 CAR-T treatment group by weight according to stratified blocked randomization. On day 4, day 8 and day 12, mice were transplanted with 1×10^7^ VEC-T cells or IL-10 CAR-T cells. The body weight of mice was monitored once a week. After two weeks of leukemia cells inoculation, in vivo imaging of the mice was performed once a week using Xenogen IVIS Spectrum and analyzed with Living Image software. Circulating VEC-T, CAR-T cells and leukemia cells were analyzed by flow cytometry. Twenty-one days after leukemia cells inoculation, mice were euthanized (another set of parallel experiments), and the liver, spleen and bone marrow of mice were separated and analyzed by pathological diagnosis.

*For murine IL-10 CAR-T in vivo*

Female C57BL/6 mice aged 8-10 week were used for in vivo experiments. Briefly, mice received 4.5Gy of sublethal total body irradiation and inoculated with 5×10^5^ MLL/AF9 cells. On the day 4 and day 8 after MLL-AF9 cells inoculation, mice were injected with 5×10^6^ mVEC-T or mIL-10 CAR-T. Twenty-one days after MLL-AF9 cells inoculation, mice were sacrificed and analyzed for immunosuppressive cells in bone marrow.

**Colony formation assay**

5×10^4^ UCB CD34^+^ cells were cocultured with CAR-T or VEC-T at E:T ratio of 1:1 for 24 h. Then, 500 CD34^+^ cells from each group were seeded in 1ml MethoCult^TM^ H4434 (Cat.# 04444, STEMCELL, Canada) in a 6-well plate. After 14 days, BFU-E, CFU-GM, and CFU-GEMM colonies were counted and recorded.

**Statistical analysis**

All experiments were performed at least in triplicate. Values were expressed as Mean±S.D. If not specifically mentioned, statistical significance of data was assessed by Student’s *t* test. A *p* value <0.05 was considered statistically significant.

**Reference**

1. Ghandi M*, et al.* Next-generation characterization of the Cancer Cell Line Encyclopedia. *Nature* 2019 May; 569(7757)**:** 503-508.

2. Chandrashekar DS*, et al.* UALCAN: A Portal for Facilitating Tumor Subgroup Gene Expression and Survival Analyses. *Neoplasia* 2017 Aug; 19(8)**:** 649-658.

3. Li S*, et al.* CD33-Specific Chimeric Antigen Receptor T Cells with Different Co-Stimulators Showed Potent Anti-Leukemia Efficacy and Different Phenotype. *Hum Gene Ther* 2018 May; 29(5)**:** 626-639.

4. Zheng W*, et al.* Regnase-1 suppresses TCF-1+ precursor exhausted T cell formation to limit CAR T cell responses against ALL. *Blood* 2021.

5. Lanitis E*, et al.* Optimized gene engineering of murine CAR-T cells reveals the beneficial effects of IL-15 coexpression. *J Exp Med* 2021 Feb 1; 218(2).

6. Wang Y*, et al.* Targeting FLT3 in acute myeloid leukemia using ligand-based chimeric antigen receptor-engineered T cells. *J Hematol Oncol* 2018 May 2; 11(1)**:** 60.
